# Distribution of sentinel nodes from parotid tumors–A feasibility study

**DOI:** 10.1002/cam4.6612

**Published:** 2023-09-30

**Authors:** Lalle Hammarstedt‐Nordenvall, Rusana Bark, Alexandra Elliot, Mathias Von Beckerath, Caroline Gahm

**Affiliations:** ^1^ Department of Head and Neck Surgery, Medical Unit Head Neck Lung and Skin Cancer Karolinska University Hospital Stockholm Sweden; ^2^ Department of Clinical Sciences Intervention and Technology, Division of Ear Nose and Throat Diseases Karolinska Institute Stockholm Sweden; ^3^ School of Medical Sciences, Faculty of Medicine and Health Örebro University Örebro Sweden

**Keywords:** neck treatment, occult methastasis, parotid tumors, parotidectomy, sentinel node (SN)

## Abstract

**Background:**

Optimum management of the N0 neck is unresolved in parotid salivary gland cancer. Sentinel node biopsy (SNB) can reliably detect microscopic lymph node metastasis and its´ clinical use is increasing for head and neck tumors. The object of this study was to establish whether the technique is applicable to detect distribution of sentinel nodes for parotid tumors.

**Materials and Methods:**

Prosepective observational study in 30 patients with benign or low‐grade T1‐T2N0 malignant tumors in the parotid gland planned for surgical treatment. Distribution of SN was detected with a preoperative ultrasound‐guided peritumoral injection with a technetium‐99 (Tc‐99 m) laballed tracer followed by a SPECT–CT and intraoperative measurement in the neck and parotidal tissue. In patients with cytologically suspected malignant tumor or highly unclerar cytology, SNB was also performed.

**Results:**

Sentinel nodes (SNs) were detected in 26/30 cases. Out of these, 7 presented with only one SN, whereas multiple sentinel nodes where detected in 19 cases. No SNs were found in neck level 1. SN was detected in level 5 independent of tumor location within the parotid gland. An intraparotidal distribution of SNs was more frequent in larger tumors.

**Conclusions:**

The use of SN‐technique in the planning of surgical treatment of parotid tumors seems feasible. It may be of clinical value for patients with parotid cancer to enable a more accurate staging and to detect occult metastasis in the SNs within the parotid as well as in the neck, enabaling the possibility to surgically remove all positive SNs at primary surgery and with reduced surgical morbidity.

## Graphical abstract

Prosepective observational study in 30 patients with T1‐T2N0 benign or low‐grade malignant tumors in the parotid gland planned for surgical treatment. Sentinel nodes (SNs) were detected in 26/30 cases, also within the parotid gland. The use of SN‐technique in the planning of surgical treatment of parotid tumors seems feasible and may enable to detect occult metastasis in the SNs within the parotid as well as in the neck.

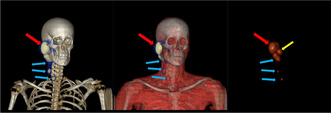

## 1 INTRODUCTION

Primary parotid gland carcinomas are rare and represents about 1%–3% of the head and neck malignancies. The disease affects almost all age‐groups and histopathology is ranging from high malignant to low malignant. Today there are 24 different histopathological malignant subtypes.^1^ Metastases in intraparotideal and/or in cervical lymph nodes are a major factor in therapy and prognosis of parotid cancer.^2^ Moreover, parotid carcinomas show a various rate of occult metastasis rate (12%–45%) mainly depending on histopathologic grade and T‐stage.^3^, ^4^, ^5^ The treatment of the neck in cN0 disease is optional and varies between centers, from a staging neck procedure when cytology indicates a high‐grade tumor, to a therapeutic neck dissection in a second procedure when the postoperative histopathologic analysis of the primary tumor is clear. Some centers recommend a watchful waiting strategy.^6^ With a varying risk of occult metastasis and thus a risk of over treatment with an elective neck dissection in cN0 disease, the extent of recommended surgical treatment is under debate. The decision to treat the neck elctively should also be based on the type of tumor histhopathplogy, which can be difficult to assess accurately with preoperative fine needle cytology (FNAC), since the cytologic diagnosis on salivary gland pathology is known to be difficult.^7^

In the last years the interest in the sentinel node (SN) technique has increased within the head and neck field and has become a routine procedure in many centers today, mainly for cancers in the oral cavity.^8^ However, the literature covering lymphatic drainage and SN‐distribution in parotid gland tumors is still sparse.^9^, ^10^, ^11^ Only one study addresses the distribution of intra‐ and periglandular SNs.^11^ Additionally, the frequency of SNs distributed to intraparotid lymph areas is unknown. Hence, there is a need for further development in this field. The aim of this prospective study was to analyze the individual lymphatic drainage pattern and SN‐distribution in patients surgicaly treated for parotid salivary gland tumors, both benign and malignant. The SN pattern distribution was assessed in regard to both parotid tissue and the neck field.

## 2 MATERIALS AND METHODS

### 2.1 Study design and eligibility criteria

In this prospective observational study, 30 consecutive patients diagnosed and treated for parotid salivary gland tumor between 2020 and 2022 at Karolinska University Hospital, Sweden, were enrolled. Patients included met the following inclusion criteria: (1) cytologically verified stage T1‐2N0 tumor or a benign tumor in the parotid salivary gland where the tumor was considered resectable, (2) swedish or english speaking with willingness to participate in the study, (3) age over 18 years, and (4) surgery with tumor excision, where preoperative SPECT–CT (single‐photon computed tomography with CT) and per‐operative gamma probe detection of the SNs could be performed. Patients previously treated with surgery or radiotherapy towards the parotid gland were excluded.

In all patients, tumor and nodal status were staged by clinical examination, computed tomography (CT) and/or magnetic resonance imaging (MRI) and fine needle aspiration cytology. In patients with cytologically suspected malignant tumor or highly unclerar cytology, SN‐biopsy (SNB) was also performed.

### 2.2 SN‐detection and surgery

Intraoperative facial nerve monitoring was performed in all patients. The removal of the tumor was preceded by a preoperative ultrasound guided peritumoral injection with a Tc‐99 m (technetium‐99) labeled tracer in ecogeneic normal salivary gland tissue superficial to intraglandular tumor. Tilmanocept (Lymphoseek, Cardinal Health) was used as a tracer until February 2021, and thereafter replaced by Nanocolloid (Nanocoll, GE Healthcare). Imaging with a strictly standardized SPECT–CT (single‐photon computed tomography with CT) was performed at earliest 1.5 h after peritumoral injection, covering the head, neck, and thorax. Surgery was performed up to 24 h after Tc‐99 m‐labeled tracer. Localisation of Tc‐99 m was intraoperatively confirmed by gamma probe (EuroProbe, Euromedical Instruments). During surgery, measurements with gamma probe of neck levels I–V both on the ipsi‐ and contralateral side were performed. Also, at the end of the surgery when the tumor was resected, the remaining of the parotid gland was rechecked with gamma probe to evaluate eventual remaining Tc‐99 m‐uptake. The following data were included in the perioperative protocol: amount of injected radionuclide (Lymphoseek/Nanocolloid), time between radionuclide injection and SPECT–CT, type of parotid surgery performed (total parotidectomy, superficial parotidectomy or parotid tailresection), perioperative localization of the tumor in the parotid (superficial lobe, deep lobe, parotid tail), persistent parotidal gamma probe signal (SN‐detection) after tumor resection (Yes/No) and in which neck levels SNs were identified with gamma probe detection (levels I–V). Parotid tail was defined according to Hamilton et al.^12^

### 2.3 Statistical analysis

Descriptive data were presented in percentages. The differences in distribution between the different subsites and T‐stage was analyzed with Fisher's exact test. A *p*‐value of <0.05 was considered significant.

## 3 RESULTS

The study included a total of 30 patients. Patients characteristics, tumor size, and tumor location are shown in Table 1. The analysis of the preoperative FNAC was interpreted as benign salivary gland tumor in 18/30 patients, a malignant salivary gland tumor in 3/30 patients and uncertain salivary gland tumor in 9/30 patients.

**TABLE 1:** Patient characteristics, tumor size, tumor location and histopathological diagnosis.

	Number of patients
Gender	
Male	18
Female	12
Age	
Min	20
Max	80
Median	53
Tumor size	
<2 cm	16
>2 cm	14
Tumor location	
Superficial parotic lobe	17
Deep parotic lobe	5
Parotic tail	8
Histopatological diagnosis	
Pleomorphic adenoma	19
Mucoepidermoid cancer (low grade)	2
Acinic cell cancer	2
Cancer ex pleomorphic adenoma	1
Salivary duct carcinoma	1
Basosquamous cancer	1
Other benign tumors	4

The preoperative FNAC diagnosis was consistent with histopathologic diagnosis in 26/30 patients and inconsistent in 4/30 patents. In three of these four patients, histopathological diagnosis was consistent as benign or malignant, but with different type of salivary gland tumor compared to the preoperative FNAC diagnosis and in one patient histopathological analysis showed an unexpected malignant diagnosis (cancer ex pleomorphic adenoma) compared to benign cytology diagnosis (pleomorphic adenoma). TNM classification within the seven patients with malignant tumors were T1N0M0 in three patients (two patients with low grade mucoepidermoid cancer and one patient with acinic cell cancer), and T2N0M0 in four patients (one patient with basosquamous cancer, acinic cell cancer, salivary duct cancer and cancer ex pleomorphic adenoma, respectively).

### 3.1 SN detection and SN distribution

**FIGURE 1:**
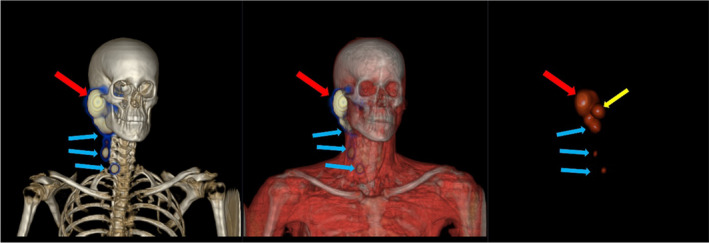
SPECT–CT showing individual lymphatic drainage to SNs from a patient with parotid tumor. Red arrow = injection site around the tumor; blue arrows = SNs in the neck; yellow arrow = intraparotid SN.

Uptake of radiotracer in radiological suspected SNs was detected on SPECT–CT in 26/30 patients. All SNs identified on SPECT–CT were also identified with gamma probe during the surgery. An example of a parotid tumor lymphatic drainage to multiple SNs (including the intraparotid tissue) on SPECT–CT is shown in Figure 1.

In 7/30 patients, only one SN was detected, while multiple SNs were detected in 14/30 patients. Larger tumors were found to have a tendency for draining to multiple SNs. In five patients with a cytologic malignant or highly unclear cytology, SNs were also excised as sentinel lymph node biopsies (SLNBs). In one of these five patients, histopathological SN analysis detected a micrometastasis. In 4/30 patients, SPECT–CT did not detect any SNs and no correlation to tumor size could be seen in these cases. The distribution of SNs are showed in Table 2.

**TABLE 2:** Distribution of sentinel nodes in regard to size and localization of the tumor in the parotid gland.

	All Sites *n* (%)	Tumor in parotid tail	Tumor in superficial Lobe	Tumor in deep lobe	Tumor ≤2 cm	Tumor>2 cm
No patients	30	8	17	5	16	14
SN level 1	0 (0)	0 (0)	0 (0)	0 (0)	0 (0)	0 (0)
SN level 2	23 (77)	7 (88)	12 (71)	4 (80)	12 (75)	11 (79)
SN level 3	2 (7)	0 (0)	2 (12)	0 (0)	1 (6)	1 (7)
SN level 4	7 (23)	3 (38)	4 (24)	0 (0)	4 (25)	3 (21)
SN level 5	7 (23)	1 (13)	5 (29)	1 (20)	4 (25)	3 (21)
SN in periparotid tissue^a^	9 (36)	1 (13)	8 (47)	NA	2 (14)	7 (64)
SN in one neck level	18 (60)	4 (50)	9 (47)	5 (100)	10 (63)	8 (57)
No SN detected	4 (13)	1 (13)	3 (18)	0 (0)	2 (14)	2 (12)

Abbreviation: SN, sentinel node.

^a^ Tumors in deep lobe excluded.

The presence of intraparotid SN could only be assessed in 25 patients, since total parotidectomy was performed in all five patients with tumor located in deep parotid lobe (Table 2). No SNs were found in neck level I, contralateral neck or in the retropharyngeal space. When comparing between tumors with diameter of >2 cm with those ≤2 cm, the larger tumors presented SNs in the intraparotid tissue in to a higher extent than the smaller tumors (7/11 vs. 2/14), however not significant. (Table 3). No difference was seen in SN distribution to one neck level regarding to tumor size.

**TABLE 3:** Presence of intraparotid sentinel node and in single neck level in relation to tumor size.

Tumor size			
	<2 cm	>2 cm	*p* ^b^
Sentinel node in intraparotid tissue^a^,% (*n*)	14 (2)	63 (7)	0.08
Sentinel node in one neck level, % (*n*)	63 (10)	57 (8)	0.56

^a^ Tumors in deep lobe excluded.

^b^ Fisher's exact test.

## 4 DISCUSSION

This study describes the individual lymphatic drainage pattern and SN distribution from patients with clinically T1‐T2N0 parotid tumors. The distribution of SNs was mainly detected within the parotid gland itself or in level II and V on the ipsilateral neck. The distribution of SNs within the parotid gland was detected regardless on where the tumor was situated in the gland, that is, intaparotid SN distribution was seen also in tumors located in the parotid tail.

Previous data on distribution of metastases from parotid tumors is mainly based on single institutional case series and are somewhat incongruent, showing differences in metastatic spread to different neck levels.^13^ In that sense, the use of SN‐technique during parotid surgery has the ability to guide both the staging of the tumor, as well as providing more accurate treatment. However, the existing data on sentinel node in parotid tumors are very sparse and today only two reports are published on sentinel node in this topic.^9^, ^11^ Our results are mainly consistent from these two series.^9^, ^11^ There was a small incongruence compared to Pan et al, where no SNs were found within the parotid gland, as well as a slightly different SN‐distribution to the neck.^9^ However, Pan et al performed the SN‐detection in local anesthesia before the parotidectomy was performed, and without prior SPECT–CT. Our findings are congruent with the seven cases described by Schilling et al, performed in a similar setting.^11^

Additionally, our findings are also in agreement with findings from Stodulski et al regarding pattern and distribution of occult metastasis from parotid cancers in a series of 66 patients with a cN0 disease treated with parotidectomy and neck dissection, where occult metastases were found in level II (80%), level III (45%) and level V (30%).^13^ Moreover, they performed a literature search with compilation of 650 cases of cN0, where 80 cases had occult metastasis mainly in level II and III but with some compiling differences.^14^ For example, in two series the risk of occult disease within level 5 was obvious with proportions of 24%–30%,^13^, ^14^ whereas in other series proportions of 0%–5% of occult metastasis to level 5 were detected.^3^, ^15^, ^16^, ^17^

Metastasis within the major salivary gland is not considered as regional disease in the TNM‐classification but refers to cervical lymph nodes.^18^ The presence of metastasis in the lymph nodes within the parotid gland is however a negative prognostic factor, with a significantly higher risk for locoregional recurrence.^19^ The distribution of intraparotidal SNs may also be dependent on where the tumor is located in the gland. In the present study, SNs in the periparotidal tissue was detected in 13% of patients who underwent parotid tailresection, compared to 47% in patients who underwent superficial parotidectomy. The limited number of patients in this study makes it difficult to draw any conclusions. Nevertheless, our results indicate that one cannot disregard from the risk of intraparotidal sentinel node involvement in tumors located also within the parotid tail. It cannot be excluded that some periparotidal SNs were missed due to shine through phenomenon, since intraoperative lymph nodes are in close spatial relation to primary tumor. However, potential periparotidal SNs located with some distance from the primary tumor are probably at a higher risk of being missed due primary resection if only partial parotidectomy is performed, Furthermore, if no areas with significant gamma probe signal are left within the parotid gland during resection of a malignant tumor, then no potential SNs should be missed but detectable in the histopathological analysis.

In the literature the recommendations on how to address the neck in patients with parotid cancer with cN0 disease varies, where the extent of neck dissection differs depending on the histopahology (high/low grade) and the size of the tumor.^3^, ^14^, ^15^, ^16^, ^17^ Adding the SNB‐technique to the parotid surgery and assessing lymphatic drainage in this group of patients may alleviate this lack of compliance. Schilling and colleagues developed a reliable method for detecting sentinel nodes and in particular intra‐ and periglandular lymph nodes.^11^

Recently, in the treatment protocol for SN‐technique in oral cavity cancer, the use of preoperative SPECT–CT has become regarded as standard of care.^8^, ^20^ In our study, the injection technique to deliver the tracer was used peritumoraly and superficial to the tumor, in accordance to the technique used in oral cavity cancers. However, it might be more accurate to regard these tumors as other gland tumors, that is, breast cancer, in which the tracer is delivered inside the tumor.^21^ There is obviously a need for further research in this field.

This study provides a baseline for the further development of sentinel node approach in salivary gland treatment. Compared to previous clinical studies on treatment of the neck in patients with metastatic tumors from the parotid gland it provides comparable data on the distribution of sentinel nodes.^3^, ^13^, ^14^, ^15^, ^16^, ^17^ It is also clear that the distribution of occult metastasis in salivary gland cancer may be heterogenous why a sentinel node approach would be appealing.

### 4.1 Limitations of the study

This prospective study includes a limited number of patients. The number of excised sentinel nodes were also low. Since the aim of this study was to analyze the individual lymphatic drainage pattern and SN‐distribution in patients surgicaly treated for parotid salivary gland tumors, we believe the results still be of value.

## 5 CONCLUSION

The use of SPECT–CT and SN‐technique to map the distribution of SNs from tumors in parotid gland may give more accurate information on the patients individual lymphatic drainage. The present findings indicate that adding the SN‐technique to the parotid surgery might be of clinical value for the patients with parotid cancer with a possibility to enable a more accurate staging.

In conclusion, larger studies are warranted, preferably in a multicenter setting to provide sufficient data for this group.

## AUTHOR CONTRIBUTIONS

**Lalle Hammarstedt‐Nordenvall:** Conceptualization (equal); data curation (equal); formal analysis (equal); funding acquisition (equal); methodology (lead); validation (equal); visualization (equal); writing – original draft (equal); writing – review and editing (equal). **Rusana Bark:** Formal analysis (supporting); funding acquisition (equal); investigation (equal); project administration (supporting); validation (equal); visualization (equal); writing – original draft (supporting); writing – review and editing (equal). **Alexandra Elliot:** Investigation (equal); visualization (equal); writing – review and editing (equal). **Mathias von Beckerath:** Investigation (equal); visualization (equal); writing – review and editing (equal). **Caroline Gahm:** Conceptualization (equal); data curation (equal); formal analysis (equal); funding acquisition (equal); investigation (equal); methodology (equal); project administration (lead); resources (equal); software (supporting); supervision (lead); validation (equal); visualization (equal); writing – original draft (equal); writing – review and editing (equal).

## FUNDING INFORMATION

Funding was provided by: The Laryngeal Foundation and the ACTA Otolaryngologica Foundation.

## CONFLICT OF INTEREST STATEMENT

The authors declare that they have no known competing financial interests or personal relationships that could have appeared to influence the work reported in this paper

## ETHICS STATEMENT

All procedures performed within the study were in accordance with ethical standards of the institutional and national research committee and with the 1964 Helsinki Declaration and its later amendments or comparable ethical standards. Written consent was obtained from all patients. The study was approved by the Head of the Department of Head‐and Neck Cancer at the Karolinska University Hospital in Stockholm, Sweden, according to approval from the National Ethical Committee (Ethical review number: 2019–05211).

## Data Availability

The data presented in this study are available on request from the corresponding author. The data are not publicly available due to Swedish laws on personal confidential information.
